# Use of probiotics and similar in pediatric patients with Type 1 Diabetes Mellitus: a systematic review

**DOI:** 10.1590/1984-0462/2024/42/2023097

**Published:** 2024-02-12

**Authors:** Luisa Pinheiro Neiva, Luiza Correia Lopez, Rafaela Orsi Pasiani, Mayco José Reinaldi Serra, Vera Esteves Vagnozzi Rullo

**Affiliations:** 1 Centro Universitário Lusíada, Santos, SP, Brazil.

**Keywords:** Glycated hemoglobin A, Diabetes mellitus type 1, Probiotics, Glycemic control, Cytokines, Hemoglobina A glicada, Diabetes *mellitus* tipo 1, Probióticos, Controle glicêmico, Citocinas

## Abstract

**Objective:**

To perform a systematic review of randomized controlled trials, evaluating the effect of probiotics, prebiotics or symbiotics supplementation on glycemic and inflammatory control in children with Type 1 Diabetes Mellitus (T1DM).

**Data source:**

The Medical Literature Analysis and Retrieval System Online (MEDLINE/PubMed), Clinical Trials, Literatura Latino-Americana e do Caribe em Ciências da Saúde (LILACS) and Scientific Electronic Library Online (SciELO) databases were searched. Randomized clinical trials of pediatric patients with DM1 using probiotics, prebiotics or symbiotics were included, regardless of year or language of publication. Studies that did not evaluate glycated hemoglobin (HbA1c) were excluded. Metabolic results (HbA1c, total insulin dose and C-peptide) and inflammatory control [interleukin-10 (IL-10), tumor necrosis factor-alpha (TNF-α) and interferon-gamma (IFN-γ)] during probiotic supplementation or similar, related to modification of the intestinal microbiota, were analyzed. PROSPERO ID: CRD42022384485.

**Data synthesis:**

Five studies were selected for a systematic review. Regarding metabolic markers, only one of the articles that analyzed HbA1c showed a significant decrease (p=0.03) in the intervention group. One study identified a reduction in the total dose of insulin and increased C-peptide levels. Regarding the evaluation of inflammatory parameters (IL-10, TNF-α, INF-γ), there were no statistical relevant modifications.

**Conclusions:**

Current data from the literature were not conclusive in identifying an improvement in glycemic control and did not observe changes in inflammatory parameters with the use of probiotics, prebiotics or symbiotics in pediatric patients with T1DM.

## RESUMO

**Objetivo:**

Realizar uma revisão sistemática de ensaios clínicos randomizados controlados avaliando o efeito da suplementação de probióticos, prebióticos ou simbióticos no controle glicêmico e inflamatório em crianças com diabetes *mellitus* tipo 1 (DM1).

**Fontes de dados:**

As bases Medical Literature Analysis and Retrieval System Online (MEDLINE/PubMed), Clinical Trials, Literatura Latino-Americana e do Caribe em Ciências da Saúde (LILACS) e Scientific Electronic Library Online (SciELO) foram pesquisadas. Foram incluídos ensaios clínicos randomizados de pacientes pediátricos com DM1 em uso de probióticos, prebióticos ou simbióticos, independentemente de ano ou idioma de publicação. Foram excluídos os trabalhos que não avaliaram hemoglobina glicada (HbA1c). Os resultados metabólicos (HbA1c, dose de insulina total e peptídeo C) e o controle inflamatório [interleucina-10 — IL-10), fator de necrose tumoral-alfa (TNF-α) e interferon-gama (IFN-γ)] durante a suplementação de probióticos ou similares, relacionados à modificação da microbiota intestinal, foram analisados. ID PROSPERO: CRD42022384485.

**Síntese dos dados:**

Cinco estudos foram selecionados para revisão sistemática. Com relação aos marcadores metabólicos, apenas um dos artigos que analisaram a HbA1c apresentou diminuição significativa (p=0,03) no grupo intervenção. Um estudo identificou redução da dose total de insulina e aumento dos níveis de peptídeo C. Quanto à avaliação dos parâmetros inflamatórios (IL-10, TNF-α, INF-γ), não houve modificações de relevância estatística.

**Conclusões:**

Os dados atuais da literatura não foram conclusivos em identificar melhora no controle glicêmico e não observaram mudanças nos parâmetros inflamatórios com o uso de probióticos, prebióticos ou simbióticos em pacientes pediátricos com DM1.

## INTRODUCTION

Type 1 Diabetes Mellitus (T1DM) is a chronic autoimmune disease characterized by the destruction of β cells, which are responsible for the production of insulin in the pancreatic islets.^1^ This hormone is responsible for the regulation of glycemic levels in the human body and, when lacking, leads to elevation and accumulation of glucose in the blood, which in the long term may bring multiple chronic manifestations, including heart failure, retinopathy, kidney failure and neuropathy.^2^

The incidence of T1DM cases has been increasing exponentially in recent decades, especially in younger people. Brazil is considered the third country with the highest number of T1DM new cases in children and adolescents per annum.^3^ In 2021, the worldwide number of individuals younger than 20 years of age with the disease was approximately 1,500,000.^4^ A cure for the disease is not yet known, and the main strategies to manage it are the understanding of its pathogenesis, development of new technologies on management and the prevention of its onset.^5^

Specific causes of how pancreatic β cells destruction occurs in T1DM are not fully understood. The most accepted explanation is the association of genetic susceptibility and environmental triggers, such as viral infections, which would promote an autoimmunity reaction in the human body.^4^

Increasingly, studies show an evident relationship of the imbalanced gut microbiota (dysbiosis) with the development of T1DM. The microbiota of patients with established β cell autoimmunity or T1DM, compared to the general healthy population, has a lower abundance of *Bifidobacterium* and *Lactobacillus* and a significant increase of *Clostridium* and*Bacteroides*, which are known to be associated with increased gut permeability and inflammation. In addition, recent studies show that children with diabetes have less variety of microbiota and small numbers of essential bacteria to maintain intestinal mucosa integrity. Consequently, intestinal epithelial barrier function decreases, contributing to the elevation of permeability and inflammation of the intestinal epithelium, which provides a release of many lipopolysaccharides (LPSs) into the circulation, promoting increased insulin resistance and deficit in glycemic control.^6–9^

There is growing evidence that the use of probiotics, prebiotics or symbiotics may play an important role in the regulation of the gut microbiota in patients with diabetes *mellitus*.^10^ Probiotics are defined as living microorganisms that, when ingested in adequate amounts, exert health benefits on the host, such as increased immunity of the organism (immunomodulation) by harmonizing immune response. They are also responsible for changing intestinal microbiota, promoting colonization resistance and suppression of pathogens.^11^ Prebiotics, on the other hand, are defined as non-viable food components that promote health benefits to the host by modulating its microbiota, supporting the immune system. Prebiotics also have nutrient absorption effects, reducing the risk of obesity and metabolic syndrome and contribute to the inhibition of carcinogenesis, especially colorectal cancer.^11,12^ Finally, symbiotics are products with probiotic and prebiotic properties, created to improve the survival of probiotics in the gastrointestinal tract.^11,13^

There is already a systematic review of the effects of probiotics, prebiotics and symbiotics in adult diabetic patients, but there is no clear evidence of the metabolic and inflammatory control exerted by them in T1DM in pediatrics.^14^ Thus, the objective of this systematic review is to evaluate the metabolic and inflammatory repercussions of the use of probiotics and similar in pediatric patients with T1DM.

## METHOD

This systematic review was conducted under the recommendations of the Preferred Reporting Items for Systematic Reviews and Meta-Analysis (PRISMA).^15^

We included randomized clinical trial studies in pediatric patients up to 18 years of age with T1DM, diagnosed through Hb1Ac greater than 6.5%. The proposed intervention was the use of probiotics, prebiotics or symbiotics. There was no restriction on the year of publication or language.

The evaluation of metabolic control was performed through the measurement of HbA1c, total daily insulin dose and C-peptide dosage. The evaluation of inflammatory control was analyzed through the measurement of IL-10, TNF-α and IFN-γ.

The databases used for study selection were Medical Literature Analysis and Retrieval System Online (MEDLINE/PubMed), Clinical Trials, Literatura Latino-Americana e do Caribe em Ciências da Saúde (LILACS) and Scientific Electronic Library Online (SciELO) until July 10, 2023. The strategy used in MEDLINE and SciELO was: (Probiotics OR Symbiotics OR Prebiotics) AND (Diabetes Mellitus, Type 1 OR Diabetes Mellitus, Insulin-Dependent OR Diabetes Mellitus, Insulin Dependent OR Insulin-Dependent Diabetes Mellitus OR Diabetes Mellitus, Juvenile-Onset OR Diabetes Mellitus, Juvenile Onset OR Juvenile-Onset Diabetes Mellitus OR IDDM OR Juvenile-Onset Diabetes OR Diabetes, Juvenile-Onset OR Juvenile-Onset Diabetes OR Diabetes Mellitus, Sudden-Onset OR Diabetes Mellitus, Sudden Onset OR Sudden-Onset Diabetes Mellitus OR Type 1 Diabetes Mellitus OR Diabetes Mellitus, Insulin-Dependent, 1 OR Insulin-Dependent Diabetes Mellitus 1 OR Insulin-Dependent Diabetes Mellitus 1 OR Type 1 Diabetes OR Diabetes, Type 1 OR Diabetes Mellitus, Type I OR Diabetes, Autoimmune OR Autoimmune Diabetes OR Diabetes Mellitus, Brittle OR Brittle Diabetes Mellitus OR Diabetes Mellitus, Ketosis-Prone OR Diabetes Mellitus, Ketosis Prone OR Ketosis-Prone Diabetes Mellitus). In Clinical Trials: probiotics|Diabetes Mellitus, Type 1. In LILACS: ((Diabetes Mellitus, Type 1)) AND ((Probiotic OR Prebiotic OR Symbiotic)).

**Figure 1:**
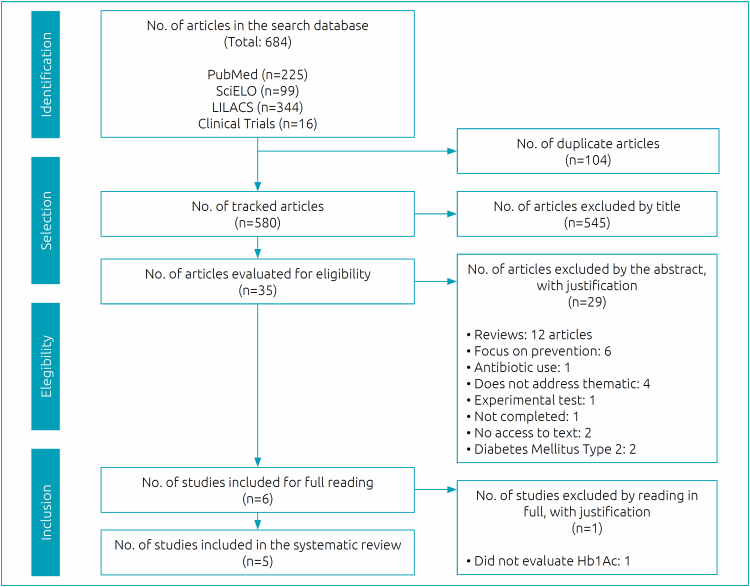
Study selection flowchart.

After applying the inclusion criteria, 684 articles were obtained. The flowchart for the selection of studies for systematic review is shown in Figure 1. Five studies were selected to be part of this systematic review.

A standardized and pre-piloted form (Excel, Microsoft Office 2017) was used to extract data from the included studies for evidence synthesis. Information was extracted from the included studies and divided into three tables:

1. Descriptive evaluation of the studies selected for the systematic review: authors’ names, year and country of publication, age studied, time of diagnosis of T1DM, which probiotic/prebiotic/symbiotic was used and doses, intervention time, total sample number (intervention versus placebo), total follow-up time;
2. Evaluation of glycemic parameters: glycated hemoglobin, total insulin dose, C-peptide;
3. Evaluation of inflammatory markers: IL-10, TNF-α and IFN-γ. Means ± standard deviations were extracted after the intervention for continuous variables related to metabolic assessment and inflammatory profile.

Such relevant data were extracted from the studies by three separate investigators (LPN, LCL, ROP). Any disagreement was resolved by a fourth independent author (MJRS). For the construction of the results table, when necessary, the conversion of the median and variance to mean and standard deviation, a methodology was used according to Hozo et al.^16^

The assessment of the risk of bias in the included studies was performed according to the revised Cochrane risk tool (RoB2),^17^ which is summarized in Table 1.^18–22^

**Table 1:** Application of the RoB2 tool to assess the risk of bias in the selected studies.

Assessed domain	I	II.1	II.2	III	IV	V	Final classification
Wang et al.^18^	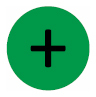	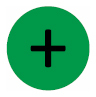	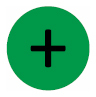	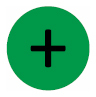	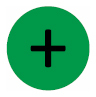	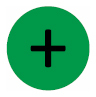	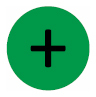
Kumar et al.^19^	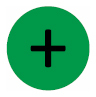	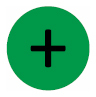	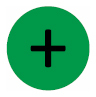	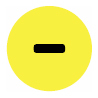	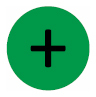	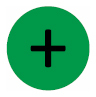	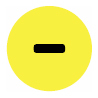
Groele et al.^20^	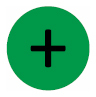	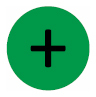	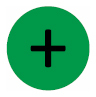	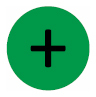	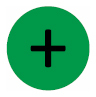	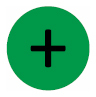	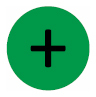
Javid et al.^21^	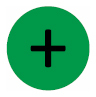	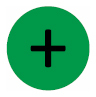	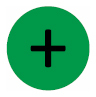	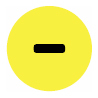	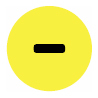	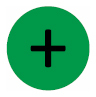	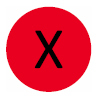
Ho et al.^22^	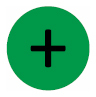	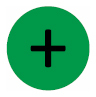	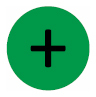	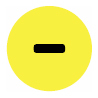	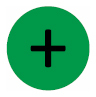	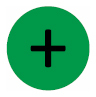	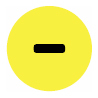

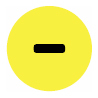
 Some concerns

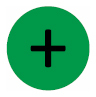

Low risk 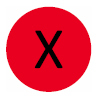 High risk

I: Bias resulting from the randomization process

II: Bias due to deviations from planned interventions

III: Bias due to lack of outcome data

IV: Outcome measurement bias

V: Bias in the selection of the reported result

## RESULTS

The evaluation of the use of probiotics, prebiotics and symbiotics in the glycemic and inflammatory control of pediatric patients with T1DM is summarized in Tables 2, 3, 4.^18–22^

**Table 2:** Descriptive evaluation of the studies selected for the systematic review.

Author and country	Age studied	T1DM diagnosis time	Probiotics or similar and doses	No. of the total sample (probiotic x placebo)	Follow-up time
Wang et al.^18^; Taiwan	6–18 years	Long-term diabetic patients who have already experienced the honeymoon period	Probiotics* **10^10^** CFU/day or placebo orally for 6 months	59 (27×32)	9 months
Kumar et al.^19^; India	2–12 years	T1DM of recent onset (within 6 months of enrollment)	Probiotics† 112.5 billion CFU/day or microcrystalline cellulose for 3 months	96 (47×49)	3 months
Groele et al.^20^; Varsovia, Poland	8–17 years	Recent diagnosis (within 60 days)	Lactobacillus rhamnosus GG and Bifidobacterium lactis Bb12 **10^9^** CFU colony or placebo (maltodextrin) orally capsule once a day for 6 months	96 (48×48)	12 months
Javid et al.^21^; Shiraz, Iran	4–18 years	At least 1 year with diagnosis of DM1	2 g symbiotic powder‡ (109 CFU) for 8 weeks	50 (25×25)	8 weeks
Ho et al.^22^; Canada	8–17 years	At least 1 year with diagnosis of DM1	8g oligofructose-enriched inulin orally once a day or placebo 3.3g maltodextrin orally once a day for 12 weeks	43 (20×23)	6 weeks

* L. salivarius subesp. salicinius AP-32, L. johnsonii MH-68 and B. animalis subesp. lactis CP-9

† Live, freeze-dried, lactic and bifidobacteria named L paracasei DSM 24733, L plantarum DSM 24730, L acidophilus DSM 24735, L delbrueckii subsp. bulgaricus DSM 24734, B longum DSM 24736, B children’s DSM 24737, B brief DSM 24732 and Streptococcus thermophilus DSM 24731

‡ Symbiotic powder: Lactobacillus sporogenes GBI-30 (probiotic), maltodextrin and fructooligosaccharide (prebiotic).

**Table 3:** Evaluation of glycemic parameters.

			HbA1c (%)		Total insulin dose (μg/mL)		C-peptide (ng/mL)	
			M±SD	p-value	M±SD	p-value	M±SD	p-value
Wang et al.^18^; Taiwan	Basal	I	9.3±0.80	0.883				
		C	9.5±1.90					
	After 6 m	I	8.5±0.90	NI				
		C	9.5±2.10					
Kumar et al.^19^; India	Basal	I	11.7±1.63	0.512	1.0±0.45	0.183	0.3±0.35	0.866
		C	11.5±2.26		0.9±0.40		0.3±0.45	
	After 3 m	I	6.8±1.46		0.6±0.31		0.5±0.40	
		C	7.4±1.46		0.7±0.40		0.5±0.35	
Groele et al.^20^; Poland	Basal	I	7.7±0.41	NI			1.0±0.27	NI
		C	8.3±0.72				1.0±0.34	
	After 6m	I	6.1±0.35	0.058			0.9±0.26	0.832
		C	6.5±0.39				0.9±0.27	
Javid et al.^21^; Iran	Basal	I	8.9±1.95	0.270*	6.4±6.32	0.810*		
		C	9.6±2.23		6.0±5.02			
	After 8 w	I	8.6±1.85	0.960*	10.9±8.20	0.150*		
		C	9.1±2.59		7.6±7.12			
		≠ I	-0.3±0.52	0.030†	4.5±4.53	0.060†		
		≠ C	-0.5±1.36		1.6±5.46			
Ho et al.^22^; Canada	Basal	I	8.0±0.82	0.854	0.9±0.25	0.928	0.3±0.75	0.928
		C	8.1±0.91		0.9±0.29		0.2±0.30	
	After 3 m	I	7.9±0.50	0.592	NI	NI	0.3±0.93	0.029
		C	8.1±0.91				0.1±0.15	

M±SD: mean±standard deviation; p: statistical difference; HbA1c: glycated hemoglobin (%); I: intervention; C: control; ≠: difference; m: months; w: weeks; NI: not informed.

* Used the independent t-test between the two groups before and after the intervention

† Analysis of covariance (ANCOVA) was performed between the two post-intervention groups after adjustment for confounding factors.

**Table 4:** Evaluation of inflammatory markers.

			IL-10		TNF-α		IFN-γ	
			M±SD	p-value	M±SD	p-value	M±SD	p-value
Wang et al.^18^; Taiwan	Basal	I			52.5±59.70	0.810		
		C			54.0±56.80			
	After 6 m	I			39.2±47.50	NI		
		C			49.3±57.20			
Groele et al.^20^; Poland	Basal	I	282.1±105.92	NI	4.6±5.29	NI		
		C	186.6±85.92		3.7±4.28			
	After 6 m	I	202.9±80.91	0.470	1.4±1.68	0.594		
		C	161.9±75.65		4.7±5.46	NI		
Ho et al.^22^; Canada	Basal	I	15.1±36.17	0.816	16.1±52.33	0.292	0.44±1.79	0.285
		C	13.0±17.91		3.9±1.75		0.02±0.04	
	After 3 m	I	18.0±10.88	0.093	17.7±3.95	0.058	0.44±0.01	0.523
		C	7.0±18.16		3.6±1.52		0.01±0.04	

M±SD: mean±standard deviation; P: statistical difference; I: intervention; C: control; m: months; n: weeks; IL-10: interleukin 10; TNF- α: tumor necrosis factor alpha; IFN-γ: interferon-gamma; NI: not informed.

The following conversions of the median and variance to mean and standard deviation were required in the Kumar et al.,^19^ study: HbA1c, total insulin and C-peptide values; and, in the Groele, Groele et al.^20^ study: HbA1c, C-peptide, IL-10 and TNF-α values, according to Hozo et al.^16^

Table 3, used to evaluate the glycemic parameters, describes five articles that evaluated HbA1c, three the total daily insulin dose and three the C-peptide, showing the results before and after the intervention. Only one of the articles^21^ that analyzed glycated Hb1Ac showed a significant decrease (p=0.03) in the intervention group. Overall, the evidence created by this review indicates that probiotics and similar do not significantly lower HbA1c. A reduction in total insulin dose and an increase in C-peptide were observed in one study.^19^ Inflammatory parameters (IL-10, TNF-alpha, INF-gamma) did not show modifications after the use of probiotics and similar with statistical relevance (Table 4) in three studies.^18,20,22^

## DISCUSSION

The current review identified a reduction of HbA1c in one of the studies analyzed and also a preservation of C-peptide levels in another study.^21,22^ No difference was found between specific inflammatory markers in three studies that investigated this association.^18,20,22^

In literature, studies have noted structural changes within the intestine of patients with type 1 diabetes, as reflected by alterations in tight junctions as well as in microvilli.^23^ Levels of zonulin, a molecule resident in tight junctions, are upregulated in patients with type 1 diabetes, and this is associated with increased intestinal permeability.^24^ Increased intestinal permeability may facilitate the absorption of antigens which can injure pancreatic β cell,^25^ which would decrease glycemic control and increase HbA1c. The evidence created by this review indicates that probiotics and similar seem to not significantly decrease HbA1c when compared to the intervention and placebo groups, since supplementation with symbiotic resulted in a significant decrease in HbA1c (p=0.03) in only one of the studies analyzed.^21^

One of the studies included in this review observed a higher serum C-peptide level in the probiotic group compared to the placebo group in a study with statistical significance (p=0.03),^22^ suggesting a persistence of pancreatic *beta* cell function. C-peptide has the function of detecting endogenous insulin secretion through *beta* cells, making it possible to identify the residual function of these cells, which is associated with better glycemic control, fewer hypoglycemic events, and a decrease in microvascular complications. In the honeymoon period in T1DM, it is possible to use C-peptide to observe whether a possible worsening in glycemic control is due to a decline in insulin secretion or, for example, poor adherence to medication.^26^ Ho et al. found no significant decrease in HbA1c with the use of prebiotics during the treatment period, but there was a preservation of C-peptide in the prebiotic group, proving to be a promising clinical marker of the prolongation of the residual function of pancreatic *beta* cells. This brings into discussion the necessity of more studies to evaluate the possibility of probiotics extending the honeymoon period due to residual insulin production for a prolonged time in pediatric patients newly diagnosed with T1DM.^22^

A study analyzed the composition and function of the intestinal microbiome in children with T1DM compared to healthy individuals. These patients with T1DM showed a decrease in the diversity and composition of the intestinal microbiome. There was an increase of *Ruminococcus* and *Bacteroides* and a decrease in *Bifidobacterium* and *Faecalibacterium*. Furthermore, as intestinal permeability is higher in T1DM and is accompanied by increased serum pro-inflammatory cytokines and LPS,^27^ probiotics may have intestinal modulation and regulation of pro-inflammatory cytokines as a mechanism of action.^28^ Therefore, their supplementation could be beneficial as they increase insulin sensitivity and reduce the autoimmune response.^29^ Two studies did not find an association of probiotics or similar with a decrease in the total daily required insulin dosage and one study found a higher insulin dose in the probiotic group. Then, probiotics or similar appear to not improve changes in total insulin daily dose.^19,21,22^

The importance of probiotics in controlling inflammation is discussed. Its consumption may be associated with reduced inflammatory responses. These health claims apparently elapse from probiotics’ ability to secrete antimicrobial substances, competing with other pathogens, strengthening the intestinal barrier, and modulating the immune system.^29^ The probiotic may decrease pro-inflammatory cytokines, such as INF-α, and may improve the expression of anti-inflammatory cytokines, such as IL-10,^30^ which was also analyzed in most of the studies included in this review, but without identifying statistical relevance. Prebiotics can influence the gut microbiota in a way that promotes the development of healthy immune signaling in gut-associated lymphoid tissue (GALT) and the mucosal immune system, altering lymphoid immune expression and decreasing the production/level of pro-inflammatory cytokines while increasing that of anti-inflammatories. This could also lead to an increase in IL-10 and TNF-α expression in Peyer’s Patches (PPs) and secretory IgA, with a simultaneous mucosal enlargement. The consumption of prebiotics (oligofructose-enriched inulin) positively influenced the intestinal microbiota, specifically increasing the number of *Bacteroidetes* and lactic acid-producing bacteria, which correlated with positive modulation of intestinal permeability and reduction of inflammation.^31^

Despite this, according to the studies analyzed in this review, it was not possible to state that the improvement of intestinal dysbiosis, using specific probiotics and prebiotics, is associated with a decline in the autoimmune response (with decreased inflammation) and intestinal integrity (through increased expression of junction proteins in the intestinal epithelium) in pediatric patients with T1DM.^18,20,22^

Our study has some limitations. Data extraction from studies is not blinded and the samples analyzed are small. In addition, different probiotics, prebiotics and symbiotics were used in different dosage regimens and duration of intervention, which makes it difficult to interpret the results. The high risk of bias identified through the evaluation of the methodological quality of the studies should also be considered.

Therefore, new studies are needed to determine whether there is a benefit from the use of probiotics and similar in glycemic and inflammatory control in T1DM in pediatric patients. We highlight the need to include in these studies larger samples, multicentricity, evaluation of other glycemic parameters, such as glycemic variability, and standardization of therapeutic regimens with probiotics and similar. In addition, larger studies that contemplate the best bacterial strain, concentration, doses and duration of treatment and also evaluate the C-peptide in newly diagnosed patients, would help to elucidate the real magnitude of this intervention.

In conclusion, in children with T1DM, the use of probiotics, prebiotics or symbiotics requires further studies to evaluate its relationship with HbA1c, total insulin dose and inflammatory markers. Current data in the literature showed no change in glycemic or inflammatory control of probiotics or similar use in pediatric patients with T1DM.
